# Assessment of the differentiation of *Sambucus nigra* plant parts using a multi-target and suspect screening by LC-HRMS and ICP-OES

**DOI:** 10.1007/s00216-025-06112-7

**Published:** 2025-09-20

**Authors:** M. Häßler, K. Wetzel, L. Warnecke, T. Niedenthal, L. Montero, J. F. Ayala-Cabrera, O. J. Schmitz

**Affiliations:** 1 Applied Analytical Chemistry, University of Duisburg-Essen, Universitätsstr. 5, 45141 Essen, Germany; 2 Forschergruppe Klostermedizin GmbH, Annastr. 26a, 97072 Würzburg, Germany; 3 Foodomics Laboratory, Institute of Food Science Research – CIAL (CSIC-UAM), Calle Nicolás Cabrera 9, 28049 Madrid, Spain; 4 Department of Analytical Chemistry, University of the Basque Country (UPV/EHU), Sarriena Auzoa, 48940 Leioa, Spain; 5 Research Centre for Experimental Marine Biology and Biotechnology, University of the Basque Country (PiE-UPV/EHU), Areatza Hiribidea 47, 48620 Plentzia, Spain

**Keywords:** Non-targeted analysis, Bioactive compounds, European plants, Phenolic compounds, Identification

## Abstract

Traditional plant medicine is among the most popular and strongest growing forms of alternative medicine. Based on ancient healing practices, it still plays a significant role in the treatment of many medical conditions. Its impact significantly influenced the creation of therapeutic products by using medical herbs in a continuously growing market. Therefore, it is important to properly characterize and differentiate medical plant parts. In this work, a multi-target and suspect screening approach for the elemental composition as well as the organic compound profile of different plant parts of *Sambucus nigra* was assessed using inductively coupled plasma-optical emission spectroscopy (ICP-OES) and liquid chromatography coupled to high-resolution mass spectrometry (LC-HRMS) to identify potential biomarkers and establish their correlations with the total phenolic content (TPC). For the elemental analysis, calcium and potassium were found as the main elements screened in the *S. nigra* plant parts, but with no correlation to the TPC content. In contrast, phosphorus showed a slight correlation with the TPC and significant correlations with concentrations of several phenolic compounds. Regarding the organic compound profile, 88 features were tentatively identified with high confidence (levels 1–3), some of them for the first time in *S. nigra*. These compounds are mainly associated with phenolic compounds such as flavonoids or cinnamic acid derivatives, among others. Moreover, 18 of these compounds were identified as potential biomarkers to differentiate between plant parts (i.e. rutin, quercetin, bergapten, etc.). These findings not only give insights into the characterization of *S. nigra* but might serve as a basis for evaluating individual substances as well as different plant parts regarding their potential hepatoprotective characteristics. The LC-HRMS method could serve as a basis for future analyses of medicinal plants, aiming for reduced analysis time, high separation performance (especially for phenolic compounds), and better result comparability.

**Graphical Abstract:**

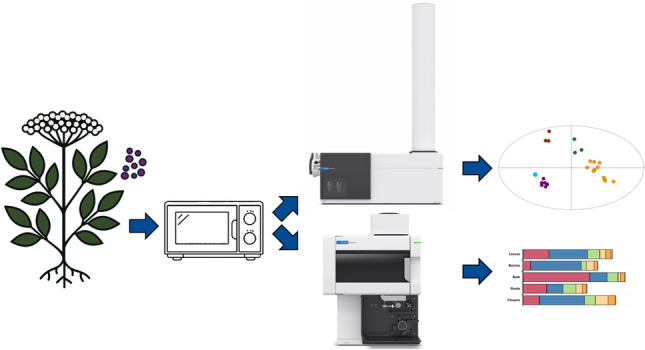

**Supplementary Information:**

The online version contains supplementary material available at 10.1007/s00216-025-06112-7.

## Introduction

European folk medicine has its roots in ancient Greek, Roman, Arabic, and monastic medical theories. Today, medicinal plants account for 40% of the world’s pharmacological products. A prominent tradition in this field is the use of native plants, known as medicinal herbs, especially for treating common illnesses [1, 2]. Notably, the use of herbal medicine among the German population increased from 52 to 70% between 1970 and 2010, leading the European market. In Germany, expenditures on herbal medicine were around 1 billion € in 2011, about 20% of the total over-the-counter drugs and reached an estimated 1.003 billion € in 2022, according to the German pharmacist association (ABDA) [3, 4].

Among these traditional herbal remedies, *Sambucus nigra *L., commonly known as elderberry, has been extensively used in botanical and pharmaceutical research due to its rich composition of bioactive compounds and significant health-promoting properties. *S. nigra* can be easily found all over the world as it grows in Europe, North America, Asia, and Northern Africa [5, 6]. Besides the location, other factors such as the level of maturity, the surrounding environment, and weather conditions influence the constituents of this plant [7].

Thus, the exploration of *S. nigra*’s phytochemical profile is not only vital for understanding its therapeutic potential but also crucial for food science and nutrition. The berries are rich in anthocyanins, flavonoids, and other polyphenols that provide potent antioxidant properties and a wide range of biological activities. These include anticancer, immunomodulatory, antibacterial, antiallergic, and antiviral effects [8, 9]. These properties support traditional applications, such as infusions for respiratory infections [10], as well as modern uses, such as natural colorants and functional ingredients in jams, jellies, yogurts, syrups, concentrated juices, and for flavoring alcoholic beverages, including bitters and wine [11]. Furthermore, the bioactive composition of S. nigra has been associated with preventing degenerative diseases, such as cardiovascular disorders, cancer, inflammatory conditions, and diabetes [12], highlighting its importance in nutrition and preventive medicine. Recent studies have significantly contributed to a better understanding of the phytochemical composition of this plant, providing an in-depth analysis of the essential oils and aroma [13], the dynamic changes in the total phenolic content (TPC), as well as anthocyanin accumulation and antioxidant activity during various growth stages and growing forms of the plant. Antioxidant activity is usually determined by DPPH free radical scavenging capacity assays with Trolox equivalents and Folin-Ciocalteu assays for the estimation of (poly)phenol content [14–17]. Several studies highlighted the predominant presence of flavonoids, particularly rutin, in elderberry fruits, underscoring their role in the plant’s antioxidant activity [14, 18]. Secondary metabolite constituents and their health benefits are also gaining more interest in the scientific community, including antioxidant, anticancer, and wound healing effects as well as antibacterial activities for capacity to inhibit the development of fungus strains and also ex situ activity for the protection of excised almond stems [18–20]. Notably, research has shown the effectiveness of extracts from *S. nigra*, particularly from its flowers, in neuroprotection and the modulation of oxidative stress in SH-SY5Y cells [21]. Although there are some studies related to berries and flowers, there is still a lack of knowledge about the potential use of other parts of the plants such as leaves or bark. Using the whole plant may be of interest in the context of maximizing resource efficiency and minimizing the environmental impact in line with the principles of circular economy and waste prevention [22–25]. Therefore, underutilized plant parts such as the leaves and bark of *S. nigra* may offer untapped potential to contribute to sustainable development and innovative health solutions. On the other hand, the origin of the plant has been usually correlated with both the elemental composition as well as the antioxidant activity, offering the potential to study potential correlations between elemental composition and antioxidant activity [26].

The aim of this work is to differentiate the various parts of the European Sambucus nigra plant based on their phenolic profiles, elemental composition, and antioxidant potential. Therefore, elemental analysis and comprehensive and detailed analysis of the phenolic compounds, known for their free radical scavenging properties and potential health benefits, have been carried out by inductively coupled plasma-optical emission spectroscopy (ICP-OES) and liquid chromatography coupled to high-resolution mass spectrometry (LC-HRMS). Additionally, metabolic relevant elements were compared with literature known phenolic activities. Combined with a suspect screening approach, differences in bioactivity between different plant parts of Sambucus nigra were discovered. Characterization of the phenolic compound profile is crucial for identifying biomarkers that could aid in the differentiation of *S. nigra* plant parts of European origin.

## Material and methods

### Materials and reagents

PTFE filters with a 0.20 µm pore size and a diameter of 13 mm provided by Macherey-Nagel (in Düren, Germany) were used. Formic acid Optima (LC/MS) with a purity of over 99% from Fisher Scientific (Schwerte, Germany), methanol of HPLC LC-MS grade from VWR Chemicals (Rosny-sous-Bois-cedex, France), and ethanol 99.7% purity (*v/v*) HPLC grade from VWR (Darmstadt, Germany) were acquired. Ammonium formate in LC-MS grade was sourced from Sigma Life Science (Steinheim, Germany) while dimethyl sulfoxide with 99.9% purity was bought from Alfa Aesar (Kandel, Germany). Ultrapure water used was produced by Satorius Arium Pro VF system (Goettingen, Germany) with 18.2 M Ω cm^−1^ conductivity. A mix of 19 analytical standards was selected to increase the identification level of selected compounds. Apigenin was provided by Cayman Chemical Company (Michigan, USA). Bergapten (99%) was obtained from Extrasynthèse (Genay, France) while chlorogenic acid (95%), curcumin (65%), DL-phenylalanine (99%), gallic acid (97.5–100%) and (±)-naringenin (95%), p-coumaric acid (98%), n-vanillylnonanamide (97%), quercetin hydrate (95%), rutin trihydrate (99%), trans-ferulic acid (99%), vanillic acid (97%), and vanillin (97%) were obtained from Sigma-Aldrich (Steinheim, Germany). Stock standards were generally prepared in methanol at 10 µM and stored at −20 °C prior to use. For phenylalanine, curcumin, bergapten, and quercetin hydrate, a 9:1 (v/v) mixture of MeOH/DMSO was used due to poor solubility.

For the elemental analysis of *S. nigra*, 65% nitric acid from Fisher Chemical (Schwerte, Germany), 30% hydrogen peroxide from AppliChem GmbH (Darmstadt, Germany), Teflon vessels from CEM (Kamp-Lintfort, Germany), ICP elemental standards (including Al, Ca, Fe, K, Mg, Na, P, S, Si, Y) at 1000 mg/L concentrations as salts dissolved in 2–3% HNO_3_, and yttrium as an internal standard from Merck (Darmstadt, Germany) were used.

### Sample collection

The dried parts of *S. nigra *L., including flowers, berries, leaves, and bark, from the years 2020 and 2021 from Poland and Bosnia were purchased (Herbathek, Berlin, Germany & Alfred Galke GmbH, Bad Grund, Germany), and from year 2023 plants were collected from different German locations (Essen, Haltern am See and Marl). After removing any extraneous matter, the plant material was washed and finely ground to a powder with a coffee mill.

The control of the harvesting process and storage conditions is highly relevant to minimizing differences in the metabolic profile of the plant. Thereby, flowers were collected at midday by cutting a branch and harvesting leaves and flowers from it. After harvesting, the flowers were dried with a desiccator in the dark for about two weeks, then separated from their clusters and stored in a dark place until extraction at room temperature. Flowers from Haltern were harvested in a park on June 13, 2023, while flowers from Marl were harvested on June 17, 2023, in a highly industrialized area. Barks and roots from the plants collected in Essen were harvested on July 31, 2023, in an urban area. For more details, see supporting information Table S1.

### Sample treatment

A microwave-assisted extraction (MAE), using a previously optimized extraction method for different parts of *S. nigra* plant [27], was used. Briefly, 125 mg of ground plant material was extracted with 5 mL of solvent. The extraction solvent consisted of different proportions of ethanol/water depending on the plant part: for flowers, leaves, and bark, the ethanol/water ratio was 40:60 (*v/v*) and for berries the ratio was 20:80 (*v/v*) [22]. The extraction was carried out using a Mars 6 (CEM, Kamp-Lintfort, Germany) microwave system at the optimal extraction conditions (55 °C and 400 W for 5 min). Afterwards, the samples were centrifuged for 10 min and filtered through a PTFE filter to remove solid particles. Filtered extracts were stored in glass vials with lids at −20 °C for short-term and −80 °C for long-term storage prior to analysis.

### Microwave digestion for ICP-OES analysis

0.5 g of the ground samples was placed in Teflon vessels previously rinsed with nitric acid. Then, 6 mL of 65% nitric acid and 2 mL of 30% hydrogen peroxide were added into the Teflon vessel and digested using a microwave. A blank was prepared in parallel, treated the same way as the sample but without the sample addition. The microwave program consisted of a 15 min ramp to a final temperature of 200 °C, while holding 600 W microwave power, followed by 15 min at a constant temperature of 200 °C. After digestion, the sample was diluted to a final volume of 50 mL with water.

### Instrumentation

The LC-MS setup consisted of a 1290 Infinity II Multisampler (G7167B), a 1290 High Speed Pump (G7120A), a 1920 MCT (G7116B), and 1290 DAD FS (G7117A) coupled to a 6546 LC/Q-TOF equipped with a DUAL-AJS ESI source (Agilent Technologies, Santa Clara, CA, USA). The chromatographic separation was achieved using a Kinetex® PFP 100 Å column (100 × 2.1 mm I.D; 1.7 µm) equipped with a 5 mm PFP guard column (Phenomenex, Torrance, CA, USA). For positive ESI ionization, a mixture of water (A) and methanol (B) both with 0.1% (*v/v*) of formic acid was used, while for negative ion mode experiments, a mixture of water (A) and methanol (B) with 5 mM of ammonium formate was used. The column was kept at a constant temperature of 50 °C and throughout the analysis and the flow was set at 0.4 mL min^−1^. The optimized gradient program was as follows: 5% B (held 1.6 min), up to 35% B at 2.6 min; up to 40% B at 6 min (held 1 min) up to 45% B at 10.5 min; up to 75% B at 11 min; up to 100% B at 17.5 min (held 2.5 min) and goes back to initial conditions for a total run time of 22 min. The injection volume was 5 µL. The QTOF operated in full scan-data dependent MS/MS acquisition mode covering a mass range from 100 to 1700 Da in both positive and negative ion modes. For the ion source, gas temperature was set to 320 °C, drying gas flow to 8 L min^−1^, nebulizer pressure to 35 psi, sheath gas temperature to 350 °C, and sheath gas flow to 11 L min^−1^. The spray voltage was adjusted to 3500 kV. The MS analyzer acquired both profile and centroid data, with a medium isolation window (~ 4 m*/z*) for MS/MS analysis. Collision energy was determined using a formula accounting for *m/z* with a slope of 4.8 and an offset of 6. The setup involved conducting MS/MS experiments on the 10 highest recorded signal intensities every 1.35 s, with a threshold of 2000 counts and a relative intensity of at least 0.01% of the total spectrum. Once a mass had been measured 12 consecutive times and an MS2 spectrum had been acquired, this specific mass was excluded from selection for 0.5 min. MassHunter Workstation LC/MS Data Acquisition 10.1 software was used for instrument control of the LC-MS setup, while MS-Dial 4.9 and MetaboAnalyst 6.0 were utilized for data processing and analysis.

Selected elements are quantified using a 5900 ICP-OES (Agilent, Santa Clara, CA, USA) instrument. The read time was set to 5 s, the RF power set to 1.3 kW, and the stabilization time of 10 s. The instrument was operated in Synchronous Vertical Dual View (SVDV) mode with a viewing height of 8 mm. The nebulizer flow rate was 0.8 L min^−1^, the plasma flow was 13 L min^−1^, and the auxiliary flow was set to 2.0 L min^−1^. The injection pump rate was set to 6.4 mL min^−1^. A screening was conducted on the plant samples to identify suitable wavelengths for element detection (see supplementary information, Table S2). The wavelengths and the concentration ranges were selected based on their peak symmetry, intensity, and their interference with wavelength from other elements (see supplementary information). To quantify the elements, calibration curves were generated using yttrium as internal standard at 0.6 µg mL^−1^. The samples were diluted in a ratio of 1:10 with 2% nitric acid, and the internal standard was added afterwards. During the analysis, 5 measurements per sample were repeated at a pump speed of 12 rpm.

### QA/QC and data processing

To ensure quality control, pooled samples from the respective plant parts were used for data filtering. QC samples were measured to monitor consistency in signal intensity across different days. The injections followed a specified sequence, which was subsequently reviewed for potential carryover. For quality assurance, the Q-TOF underwent daily mass calibration with ESI-L low concentration tune mix (Agilent, Santa Clara, CA, USA) and blanks were analyzed between each plant part sample, with triplicate measurements performed to ensure accuracy and repeatability. Before applying the suspect screening to the workflow, it was validated with the determination of the limit of identification (LOI). The LOI serves as a critical benchmark in the qualitative assurance of HRMS methodologies, showing the minimum quantity of a molecule that can be confidently identified [28]. The application of LOI in global suspect screening analysis practices exhibits a lack of standardized protocols, with multiple approaches and criteria in its determination [28–30]. In this work, analytes were only classified as “identified” if tandem MS spectra were obtained. To estimate the LOI, standards were diluted to different concentrations (10 µM, 5 µM, 1 µM, 0.5 µM, 0.1 µM, 0.05 µM, 0.01 µM, and 0.005 µM) and analyzed in triplicate by LC-HRMS.

Suspect compounds underwent several data filtering steps. First, all features that showed no intensity in one of the QC samples were filtered out. Then, all data with a relative standard deviation (RSD) higher than 35% within the QC pool samples were eliminated. The third filter step eliminated all features for which no MS2 spectra was recorded. Further, all data to which no molecular formula could be assigned were sorted out. This resulted in the elimination of all “unknowns” or level 5 features [31]. To increase the confidence of the annotations (up to level 2a or 3), experimental MS/HRMS spectra were compared with MassBank of North America (MoNA) with a required minimum mass spectral similarity of 85%. Finally, if the standard was available, upgrade to level 1 was achieved for matching retention time (± 1 min) and MS/HRMS spectra (> 85%). Estimated anthropogenic compounds were eliminated afterward. For data treatment of tentatively identified compounds, MetaboAnalyst software was used for Pareto scaling and normalization by sum to improve the reliability of the statistical data analysis [32, 33].

## Results and discussion

### Validation of the elemental analysis by ICP-OES

Accurate and comparable results require reliable elemental analysis. The objective of the experiment was to validate the analytical method by establishing concentration ranges. The establishment of these ranges was expected to enable accurate calibration. To validate the elemental analysis, a preliminary screening to estimate the concentration range for the samples was conducted. Based on these results, external calibration was performed and quality parameters were determined (supplementary information, Table S3). The limits of detection (LOD) and limits of quantification (LOQ) calculated as 3 and 10 times the signal-to-noise ratio for the ten analyzed elements ranged from 3.9 µg·L^−1^ to 129 µg·L^−1^ and from 10 µg·L^−1^ to 430 µg·L^−1^, respectively. The detection capability is comparable to that of other studies on plant samples utilizing green extraction methods [34].

### Validation of target and suspect chemical characterization workflows for the LC-HRMS analysis of main compounds present in *S. nigra*

**Fig. 1:**
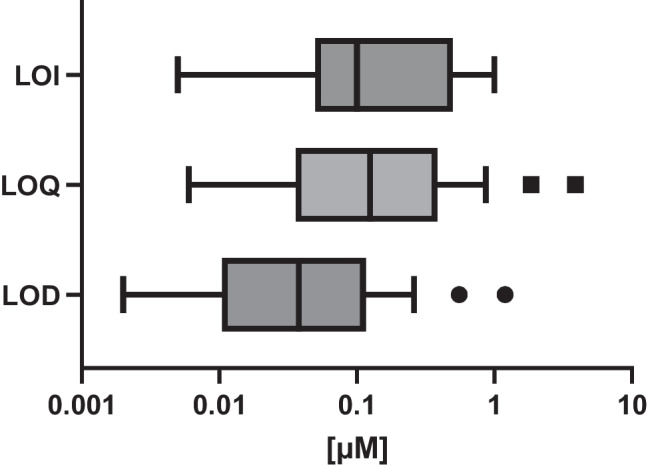
Analytical quality figures of merit box and whiskers plot

In order to ensure the reliability of the non-target screening workflow, it was crucial to evaluate its detection capabilities and overall analytical performance. The limits of detection, quantification, and identification serve to define the sensitivity and confidence of compound identification. The validation of the non-target screening workflow was achieved through the determination of its detection capability. Figure 1 shows the distribution of LODs, LOQs, and LOIs obtained for the target compounds. The selection of these compounds was made on the premise that they had been identified as level 3 during the workflow. The LOIs throughout the study were primarily established as the lowest concentration where MS/MS spectra were acquired, ensuring a confident identification [29, 30]. Figure 1 illustrates the comparability and correlation between these limits, being notable that, overall, the LOI approximately corresponds to the LOQ. Quercetin and Myricetin are not properly validated because of the low ionization efficiency by LC-ESI-HRMS. Therefore, they were left out for the box and whisker plot.

For target analysis, the method showed a good linearity (R^2^ > 0.948) along the working range (> LOD), see Table 1. Besides, precision, determined as the relative standard deviation (n = 3), and trueness, calculated as the relative error between the experimental and the calculated concentration, were lower than 30% and 41% at 1 µM and 12% and 46% at 10 µM, respectively.

**Table 1:** Assignment of LOD, LOQ and LOI for representative target compounds found in *S. nigra* and comparison of data for calculating validation parameters based on LOD calibration curves for different standards measured in positive mode. ^a^ shows medium level at 1 µM and ^b^ high level at 10 µM

Summary	LOD	LOQ	LOI	Sensitivity	Linearity	Precision		Trueness		RT	Mass	Adduct ion
	(µM)	(µM)	(µM)	Slope	R^2^	%RSD medium^a^	%RSD high^b^	%RE medium^a^	%RE high^b^	min	(pos)	
Apigenin	0.065	0.218	0.500	3,630,344	0.987	15	3	0	−46	12.0	271.0607	[M+H]^+^
Bergapten	0.002	0.007	0.010	208,226	0.999	4	2	−12	5	11.8	217.0507	[M+H]^+^
Caffeic acid	0.095	0.315	0.100	135,306	0.999	10	6	−13	3	3.6	163.0390	[M-H_2_O+H]^+^
Catechin hydrate	0.040	0.134	0.050	6,062,784	0.977	13	3	−1	−48	3.3	291.0862	[M+H]^+^
Chlorogenic acid	1.2	3.9	1.000	984,060	0.999	4	3	0	−22	3.6	355.1032	[M+H]^+^
Coumaric acid	0.261	0.869	0.500	154,218	0.996	7	1	0	−26	5.2	147.0447	[M-H_2_O+H]^+^
Coumarin	0.036	0.121	0.050	1,357,639	0.985	4	6	−30	−21	3.9	147.0444	[M+H]^+^
Curcumin	0.010	0.035	0.500	462,577	0.994	3	4	−41	11	12.4	369.1331	[M+H]^+^
Ferulic acid	0.137	0.457	0.500	703,473	0.997	7	1	0	−9	4.7	177.0552	[M-H_2_O+H]^+^
Gallic acid	0.557	1.855	1.000	42,511	0.991	7	6	−8	0	6.6	171.0286	[M+H]^+^
Myricetin	3.1	10.3	5.000	64,995	0.998	7	5	−34	3	7.3	319.0453	[M+H]^+^
Naringenin	0.011	0.037	0.050	517,640	0.948	30	5	0	−34	11.9	273.0772	[M+H]^+^
Phenylalanine	0.084	0.279	0.100	540,714	0.998	0	12	−38	−8	1.9	166.0867	[M+H]^+^
Quercetin	11.9	39.8	> 10	85,916	0.982	14	3	1	−35	10.3	303.0508	[M+H]^+^
Rutin	0.038	0.125	0.100	x	x	18	3	x	x	5.4	611.1629	[M+H]^+^
Tryptophan	0.034	0.112	0.500	1,989,499	1.000	5	1	11	0	3.5	205.0976	[M+H]^+^
Vanillic acid	0.002	0.006	0.005	241,386	0.998	13	6	−11	3	12.0	137.0603	[M+H-O_2_]
Vanillin	0.019	0.063	0.050	x	x	16	8	x	x	4.1	153.0549	[M+H]^+^

### Analysis of samples

#### Element distribution of European *S. nigra*

**Fig. 2:**
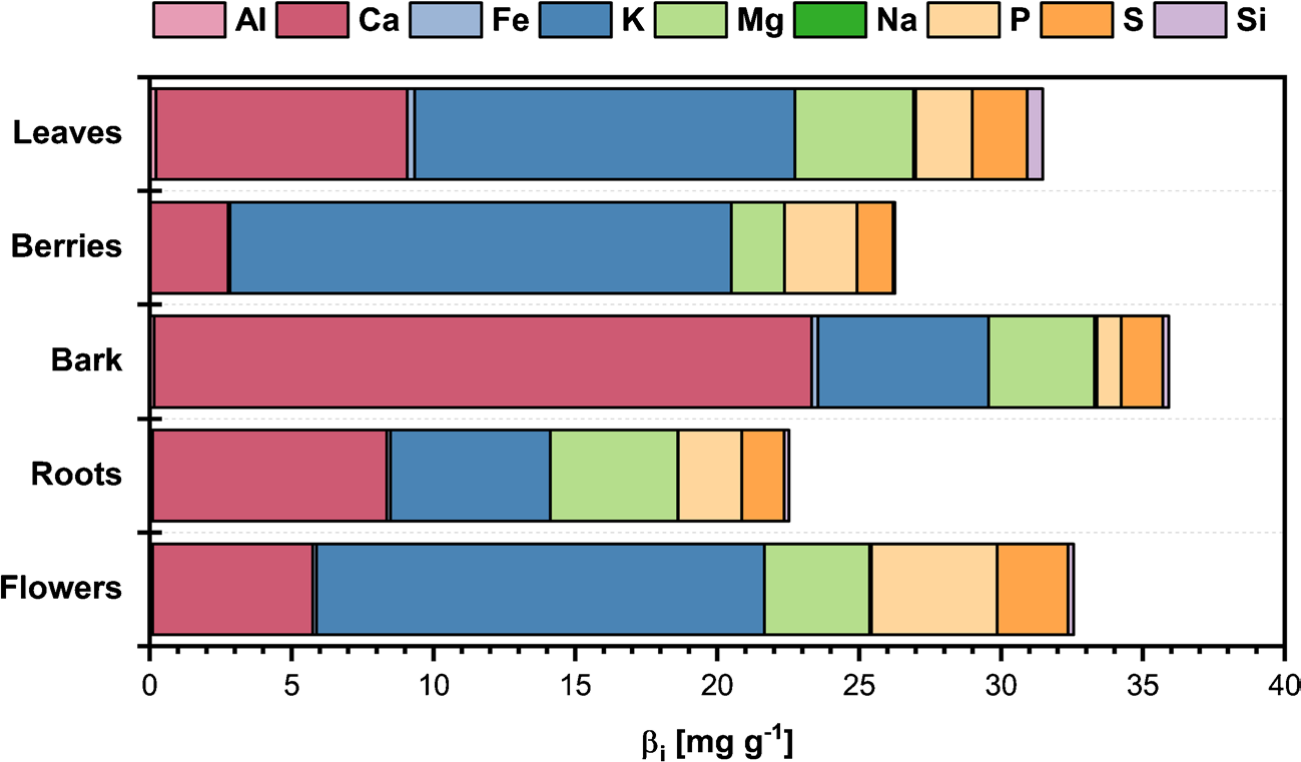
Average element distribution in % of total value for the respective *S. nigra* plant parts

The elemental distribution and total phenolic content (TPC) of Sambucus nigra were analyzed in roots, bark, leaves, flowers, and berries collected from various European regions. The targeted elemental analysis focused on physiologically relevant elements, while the TPC measurements aimed to explore potential links between mineral composition and bioactivity across plant parts and locations. As shown in Fig. 2, the average distribution of elements varies significantly between different plant parts, as for location between the flowers only slight changes in K and S concentrations are observed (see supplementary information, Table S4). In general, Ca, K, P, Mg, and S showed the highest concentration differences within the plant parts. Calcium was abundant in roots, bark, and leaves, especially shape defining plant parts, suggesting its role in structural support [35]. Potassium showed the highest concentrations in flowers and berries, likely supporting fruit formation and water transport, as already seen in diverse plants [36]. Magnesium was prominent in flowers and leaves, indicating roles in photosynthesis and enzyme activation [37], while its high concentration in roots suggests essential functions in enzymatic processes, structural stability, nutrient transport, and chlorophyll synthesis, as it is a building block of it [29, 38]. Phosphorus is crucial for DNA/RNA synthesis [39], the highest concentration is found in flowers and fruits and also with higher levels in roots and leaves, indicating roles in nutrient uptake, root development, and energy transfer [39, 40]. Sulfur was highly concentrated in leaves and flowers, supporting protein synthesis and enzyme activation [41].

It has been observed that variations in soil and environmental conditions may influence element concentrations [42]. These trends provide valuable insights into the physiological needs and environmental interactions of the various parts of the *S. nigra* plant. Therefore, the elemental composition of *S. nigra* plants from different geographical origins was analyzed. Within the different European samples, the main difference was found in bark and flower extracts. Especially potassium and sulfur varied between the flowers, which indicates differences within the soil of the plants. The bark from Essen shows elevated levels of aluminum, calcium, and iron, indicating this plant’s need for structural support. Aluminum could also be an indicator of possible contamination, as the wild-grown samples from Essen are harvested in urban areas [43]. The concentration of each element in the different parts of the analyzed plants is shown in Table S4.

**Fig. 3:**
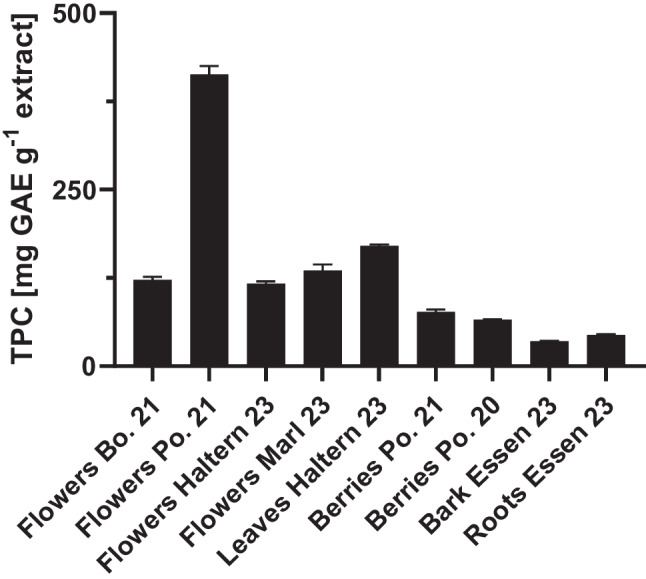
TPC in *S. nigra* plant parts from different European countries (see supplementary information Table S1)

Further, Wetzel et al. [27] showed that *S. nigra* flowers exhibit the highest total phenolic content, followed by berries, with the bark showing the lowest bioactivity. Interestingly, the phenolic content could be correlated with the antioxidant activity. In this work, the TPC of the extracts obtained for each part of *S. nigra* was measured. In Fig. 3, TPC results for the plant extracts are shown. These results show a similar trend to those already published in the literature [17, 18, 27]. Interestingly, structural support elements like calcium appear to have an inverse relationship with bioactivity, as indicated by the high levels of calcium (63%) in the bark and (37%) in the roots. After statistical analysis, the TPC showed a slight but not significant correlation (Pearson Correlation Coefficient (PCC)) with the concentration of phosphorus (0.51) and silica (0.55), whereas there was no significant correlation with the concentrations of other elements. Due to relatively low PCC values, this could be an indication that these elements do not have a primary influence on the total phenolic content within the plant parts.

#### LC-HRMS non-target chemical characterization of organic compounds

**Fig. 4:**
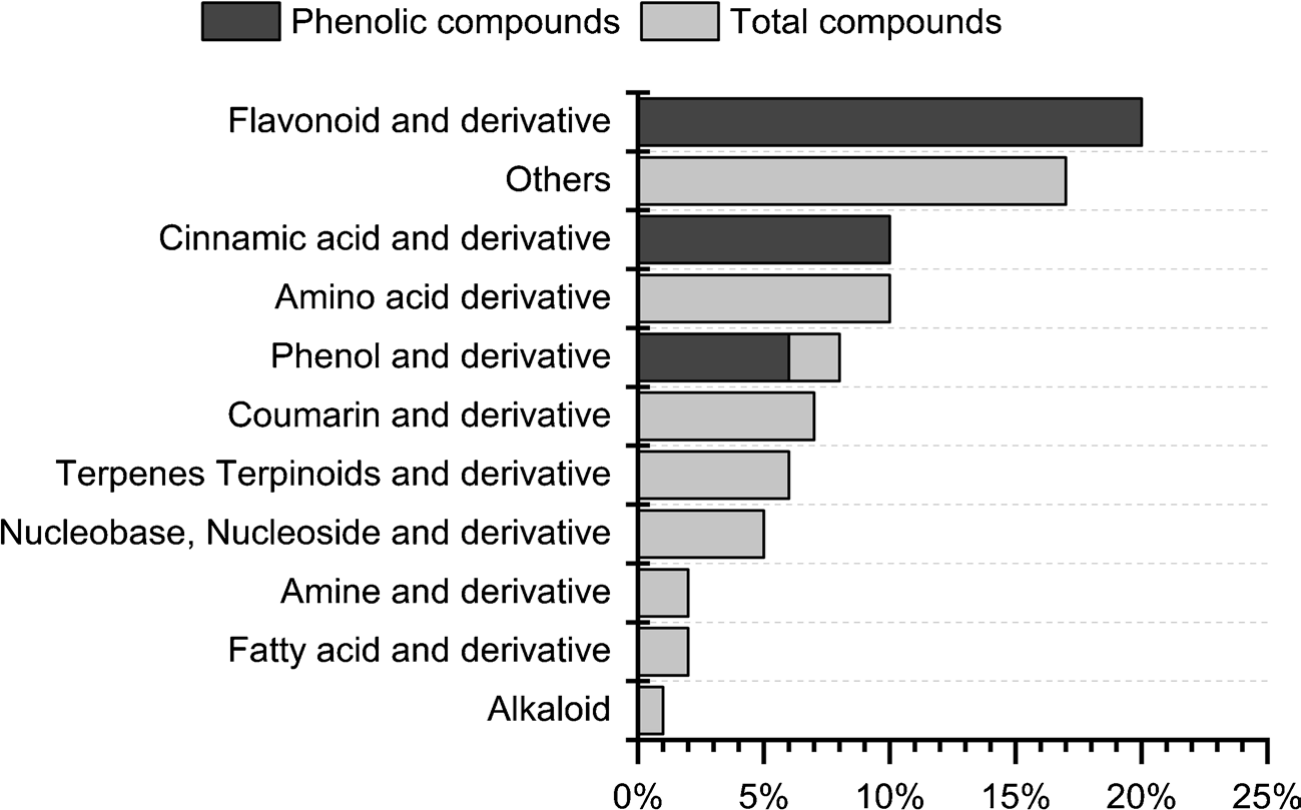
Compound classes of the identified compounds (levels 1–3) in all plant parts, with phenolic compounds within these groups marked in light gray

A non-targeted chemical characterization of the secondary metabolites present in the extracts obtained from the nine samples was analyzed. This leads to 164,821 detected features. The filtering process resulted in 497 features for all plant parts. Afterwards, principal component analysis (PCA) and partial least squares discriminant analysis (PLS-DA) were carried out (see supplementary information Figure S1) to identify features that can potentially differentiate plant parts. The top 200 distinguishing features in positive ion mode, together with an additional 41 features identified in negative ion mode, were examined. After removal of features tentatively identified as anthropogenic compounds, as well as the merging of isomeric features, the final dataset comprised 121 individual features, 88 (73%) being classified as level 3 or higher, as shown in supplementary information Table S5. The main compound classes tentatively identified are summarized in Fig. 4. Thirty-six of those could be classified as phenolic compounds.

Flavonoids and cinnamic acids are both subclasses of phenolic compounds; both contribute to plant defense mechanisms and to their associated health benefits, such as their anti-inflammatory activities [7].

Among all the compounds tentatively identified, 18 could be classified at level 1, 20 at level 2, and 50 at level 3 according to the Schymanski scale [31]. Compounds identified as level 1 were quantified in the respective plant parts (see supplementary information, Table S6). Many of the compounds identified at level 1 could be classified as phenolic compounds. Some of these were rutin and its precursor quercetin, which have been strongly associated with antioxidant activities [44, 45].

Among the level 1 identified compounds, it is important to highlight that, to the best of our knowledge, this is the first time that bergapten, coumarin, curcumin, and vanillin are identified in *S. nigra*.

**Fig. 5:**
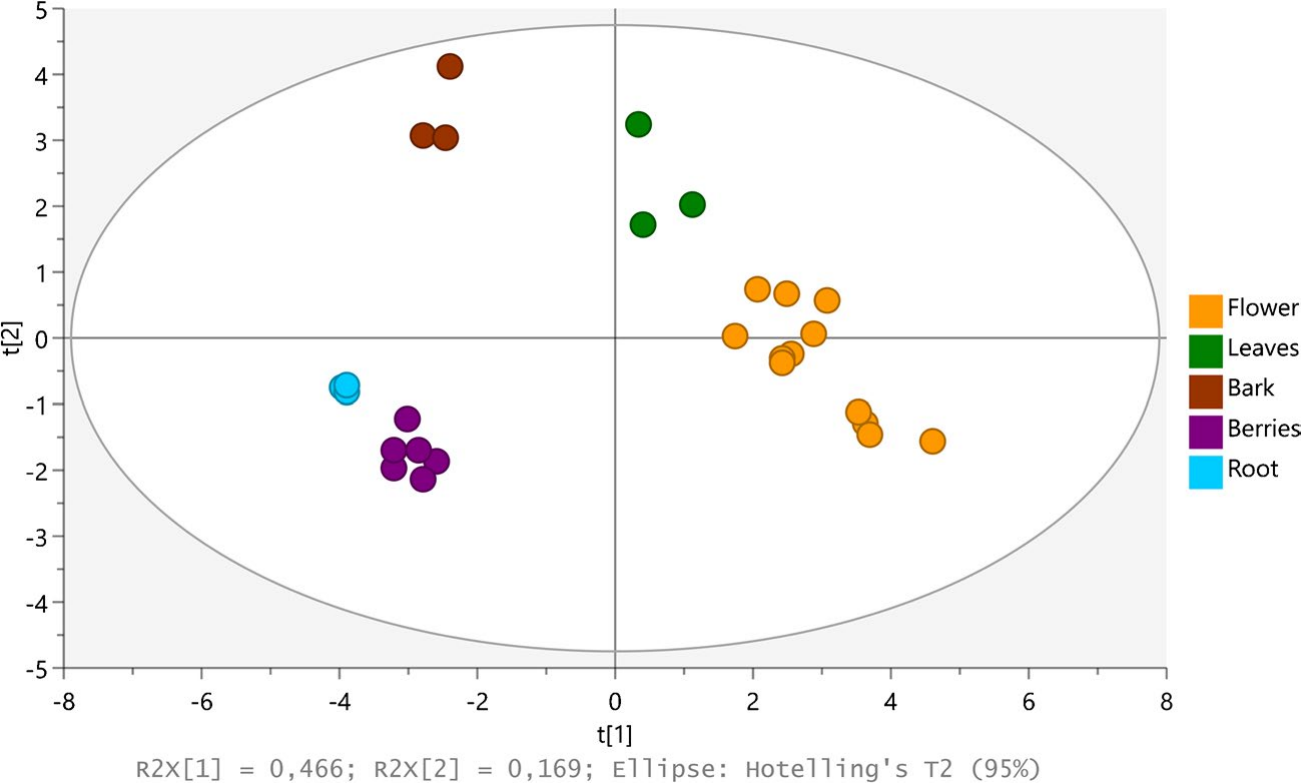
PCA of plant parts using concentration of level 1 identified compounds as biomarkers

In order to evaluate the most representative compounds from each plant part, a PC analysis of those 18 compounds identified at level 1 was performed. Based on the PCA, the 18 level 1 identified compounds showed the potential to be used as biomarkers to differentiate plant parts, as seen in Fig. 5. These biomarkers could be used for the identification of the plant parts used for the preparation of different *S. nigra*-based herbal medicines.

Further, the TPC was correlated with the quantified phenolic compounds showing a high and significant correlation (PCC 0.62, *p*-value 0.073). The correlation can be increased to a PCC of 0.91 (*p*-value of 0.00062) by excluding flower samples obtained from Poland. This suggests that the Polish samples have a unique composition and differ from the other samples, possibly due to specific phytochemical differences or environmental factors influencing phenolic compound production.

The PCC for chlorogenic acid, a phenolic compound highly associated with free radical scavenging abilities and that was observed at high concentration in the samples, was at 0.88 with a *p*-value of 0.00016. Rutin (PCC at 0.94; *p*-value 0.00015), a flavonoid, directly correlates with TPC and was found in flowers and leaves, and contributes significantly to the elevated TPC in berries. Caffeic acid (PCC 0.89; *p*-value 0.0013) was a compound that presented high concentration in flowers, leaves, and bark samples; therefore, it further contributed to the TPC value, as well as vanillic acid (PCC 0.83; *p*-value 0.0054), present in flowers and leaves. However, ferulic acid (PCC 0.04), primarily found in bark and leaves, gallic acid (PCC −0.20), most abundant in berries, and coumaric acid (PCC 0.20) deviate from this general correlation. Only coumaric acid has another significant correlation with a PCC of 0.67 and a *p*-value of less than 0.05. The other compounds have no significant correlation. These findings suggest that while most phenolic compounds contribute positively to the total phenolic content in *S. nigra*, certain compounds such as gallic and ferulic acid may play a more context-dependent or structurally distinct role within the plant’s phenolic network, possibly due to differences in biosynthesis pathways or compartmentalization; also, the correlation could change under different extraction conditions.

### Correlation between elemental composition and phenolic compound profiles

Moreover, the concentration of elemental composition was also correlated with the sum of concentrations of compounds identified as level 1, showing a strong and significant correlation with phosphorus (PCC 0.71, *p*-value 0.030) and a non-significant correlation with sulfur (PCC 0.54, *p*-value 0.16). When looking at individual compounds in Table 2, vanillin and ferulic acid show significant correlations with Ca and Fe, displaying an opposite trend compared to previous findings. Interestingly, the gallic acid concentration is significantly negatively correlated with magnesium levels, indicating an almost inverse relationship. While apigenin, coumaric acid, naringenin, rutin, and vanillic acid correlate with phosphorus, as expected due to the TPC correlation, which might indicate the location of synthesis for e.g., pentose phosphate pathway, shikimic acid pathway, and other energy transfer processes with ATP and NADP^+^ for the polyphenolic compounds within the plant [46, 47].

**Table 2:** Pearson correlation coefficients and *p*-values between quantified amount of the identified compounds (level 1) and the quantified elements. Significant correlations with a Pearson correlation coefficient higher than 0.7 are highlighted

	Al		Ca		Fe		K		Mg		Na		P		S		Si	
	Pearson	*p*-value	Pearson	*p*-value	Pearson	*p*-value	Pearson	*p*-value	Pearson	*p*-value	Pearson	*p*-value	Pearson	*p*-value	Pearson	*p*-value	Pearson	*p*-value
Apigenin	−0.01	0.98	−0.28	0.47	−0.16	0.67	−0.10	0.80	0.23	0.54	0.15	0.71	**0.86**	**0.0026**	0.27	0.49	−0.03	0.94
Bergapten	0.68	0.045	0.30	0.43	0.41	0.28	−0.39	0.31	0.45	0.22	0.56	0.12	−0.07	0.86	−0.60	0.089	0.60	0.086
Caffeic acid	0.34	0.37	−0.14	0.72	0.16	0.69	0.34	0.37	0.37	0.32	0.49	0.18	0.58	0.10	0.61	0.08	0.48	0.19
Catechin hydrate	0.32	0.40	0.21	0.58	0.22	0.57	0.16	0.69	0.41	0.27	0.52	0.15	0.43	0.25	0.32	0.41	0.14	0.71
Chlorogenic acid	0.32	0.40	−0.15	0.70	0.14	0.71	0.34	0.37	0.37	0.32	0.48	0.19	0.59	0.10	0.63	0.068	0.46	0.22
Coumaric acid	0.08	0.83	−0.27	0.48	−0.13	0.75	0.37	0.33	0.34	0.37	0.14	0.71	**0.77**	**0.02**	0.54	0.13	0.13	0.74
Coumarin	0.05	0.90	0.15	0.69	0.19	0.63	−0.27	0.48	0.12	0.75	**0.88**	**0.0016**	0.51	0.16	0.63	0.068	−0.10	0.80
Curcumin	**0.86**	**0.0026**	0.50	0.17	**0.76**	**0.017**	−0.22	0.58	0.49	0.18	0.58	0.10	−0.57	0.11	−0.15	0.70	**0.94**	**0.0002**
Ferulic acid	**0.73**	**0.026**	**0.81**	**0.0078**	**0.91**	**0.0006**	−0.17	0.66	0.27	0.48	**0.86**	**0.0028**	−0.42	0.26	0.23	0.54	0.57	0.11
Gallic acid	−0.74	0.022	−0.44	0.23	−0.61	0.078	0.28	0.47	**−0.95**	**0.0001**	0.63	0.067	0.02	0.96	−0.19	0.62	−0.63	0.07
Naringenin	−0.22	0.57	−0.31	0.42	−0.23	0.56	0.08	0.83	0.08	0.84	0.06	0.87	**0.86**	**0.0028**	0.68	0.042	−0.22	0.57
Phenylalanine	−0.15	0.70	−0.44	0.23	−0.32	0.40	0.09	0.81	−0.32	0.39	0.49	0.18	0.57	0.11	−0.03	0.94	0.0025	0.99
Rutin	0.10	0.80	−0.46	0.21	−0.17	0.67	0.29	0.44	0.20	0.61	0.23	0.55	**0.76**	**0.017**	0.43	0.25	0.32	0.41
Tryptophan	0.67	0.05	0.30	0.43	0.55	0.13	−0.17	0.66	0.42	0.25	**0.86**	**0.0032**	0.25	0.52	0.15	0.70	0.64	0.063
Vanillic acid	0.06	0.88	−0.57	0.11	−0.26	0.50	0.19	0.63	0.24	0.54	−0.08	0.84	**0.71**	**0.034**	0.18	0.64	0.32	0.41
Vanillin	0.47	0.20	**0.93**	**0.0002**	**0.78**	**0.013**	−0.33	0.39	0.01	0.98	0.68	0.044	−0.65	0.057	−0.18	0.64	0.14	0.71

## Conclusion

The differentiation of *S. nigra* plant parts has been achieved by using a LC-HRMS approach combined with ICP-OES analysis. The elemental distribution within *S. nigra* varies significantly across different plant parts. Flowers and berries exhibit high potassium concentrations. Aluminum is more concentrated in leaves, bark, roots, and flowers, while its content is reduced in berries. Calcium is enriched in roots, bark, and leaves, while iron is uniformly distributed, only slightly less concentrated in berries. Magnesium is abundant in flowers and leaves, whereas phosphorus is more concentrated in roots and leaves. Similarly, sulfur levels are higher in leaves and flowers. Regional variations, such as differences in potassium and sulfur levels between flower samples, suggest soil and environmental influences. For the non-targeted identification of metabolites present in the sample, 18 compounds were identified at level 1, 20 at level 2a, 50 at level 3, and 33 at level 4. When comparing the results of the quantified level 1-identified compounds, a strong correlation (PCC 0.91, p = 0.0006) between total phenolic compound content (TPC) and quantified concentrations is observed, suggesting a unique phytochemical composition due to specific environmental factors or distinct biosynthetic pathways. Several phenolic compounds, i.e., rutin, chlorogenic acid, caffeic acid, and vanillic acid, exhibited a high correlation with TPC. Phenolic compounds significantly contribute to the antioxidant capacity of the samples. In contrast, ferulic acid and gallic acid deviate from this trend, showing no significant correlation with TPC. An analysis of elemental composition reveals no significant correlation between TPC and the measured elements. A significant correlation is observed between phosphorus and the sum of the intensities of level 1 phenolic compounds (PCC 0.71, p = 0.03), notably apigenin, coumaric acid, naringenin, rutin, and vanillic acid. Vanillin and ferulic acid show significant correlations with Ca, Fe, and Na, which indicate that they are involved in different metabolomic activities than most of the polyphenols. The results obtained can serve as a basis for further research on the identified compounds and may provide insights into plant characterization, metabolic pathways, and potential medical applications of *S. nigra*.

## Supplementary Information

Below is the link to the electronic supplementary material.

ESM 1 (DOCX 1.25 MB)

## Data Availability

All data are available to share upon request.
