# Supplementary material for: Assessment of the differentiation of Sambucus nigra plant parts using a multi-target and suspect screening by LC-HRMS and ICP-OES
Source: Anal Bioanal Chem. 2025 Sep 20;417(27):6209–20. doi: 10.1007/s00216-025-06112-7 (PMC12583296; doi:10.1007/s00216-025-06112-7)
Supplement: Supplementary file 1 — (DOCX 1.25 MB) [file 216_2025_6112_MOESM1_ESM.docx]

**Assessment of the differentiation of *Sambucus nigra* plant parts using a multi-target and suspect screening by LC-HRMS and ICP-OES**

M. Häßler^a^, K. Wetzel^a^, L. Warnecke^a^, T. Niedenthal^b^, L. Montero^a,c^, J. F. Ayala-Cabrera^a,d,e^, O. J. Schmitz^a*^

^a^Applied Analytical Chemistry, University of Duisburg-Essen, Universitätsstr. 5, 45141 Essen/Germany

^b^Forschergruppe Klostermedizin GmbH, Annastr. 26a, 97072 Würzburg, Germany

^c^Foodomics Laboratory, Institute of Food Science Research – CIAL (CSIC-UAM), Calle Nicolás Cabrera 9, 28049 Madrid, Spain

^d^Department of Analytical Chemistry, University of the Basque Country (UPV/EHU), Sarriena Auzoa, 48940 Leioa, Spain

^e^Research Centre for Experimental Marine Biology and Biotechnology, University of the Basque Country (PiE-UPV/EHU), Areatza Hiribidea 47, 48620 Plentzia, Spain

*Corresponding author mail: [oliver.schmitz@uni-due.de](mailto:oliver.schmitz@uni-due.de)

**Supplementary information**

**ORCID:**

M. Häßler: 0009-0003-5757-4208

K. Wetzel: 0009-0006-7572-3061

L. Montero: 0000-0003-1811-0913

J. F. Ayala-Cabrera: 0000-0002-4145-7731

O. J. Schmitz: 0000-0003-2184-1207

**Sample materials**

Table S1: Detailed origin of the different S. Nigra plant samples

| **Plant parts** | **Sample name** | **Place & Year of harvest** | **Provider** | **condition** | **Origin** |
| --- | --- | --- | --- | --- | --- |
| Berries | SNB Herbathek | Poland (2021) | Herbathek | Dried^a^, Whole fruits | Agriculture |
| Flowers | SNF Herbathek | Bosnia (2021) |  | Dried^a^ | Agriculture |
| Bark | SNK Herbathek | Poland (2021) |  | Dried^a^, precrushed | Agriculture |
| Bark | SNK Essen | Essen (07’2023) | Marvin Häßler | Dried^b^ | Urban Area |
| Root | SNR Essen | Essen (07’2023) | Marvin Häßler | Dried^b^ | Urban Area |
| Flowers | SNF Haltern | Haltern am See Germany (06’2023) | Lennard Warnecke | Blooming and wilted flowers, dried^b^ | Park |
| Flowers | SNF Marl | Marl Germany (06’2023) | Lennard Warnecke | Blooming flowers, dried^b^. | Parking lot (near airfield) |
| Leaves | SNL Haltern | Haltern am See Germany (06’2023) | Lennard Warnecke | Dried^b^ | Park |
| Flowers | SNF Galke | Poland (2022) | Alfred Galke GmbH | Dried^a^ | Agriculture |
| Berries | SNB Galke | Poland (2020) |  | Dried^a^. Whole fruits | Agriculture |

^a^purchased samples were airdried

^b^collected samples were dried in a desiccator protected from light at room temperature

All samples were first screened to select suitable wavelengths.

The following wavelengths were selected based on good peak symmetry and intensity:

Table S2: Selected wavelengths for elemental analysis using ICP-OES in S. nigra plant samples.

| Element | Wavelength | Concentration Range |
| --- | --- | --- |
|  | [nm] | [ppm] |
| Aluminum (Al) | 396.152 | 0 - 4 |
| Calcium (Ca) | 370.602 | 0 - 100 |
| Iron (Fe) | 238.204 | 0 - 2 |
| Potassium (K) | 766.491 | 0 - 100 |
| Magnesium (Mg) | 277.983 | 0 - 70 |
| Sodium (Na) | 589.592 | 0 - 5 |
| Phosphorus (P) | 213.618 | 0 - 60 |
| Sulfur (S) | 180.669 | 0 - 20 |
| Silicon (Si) | 251.611 | 0 - 10 |
| Yttrium (Y)^a^ | 371.029 | 0.6 |

^a^Internal standard

Table S3: Method validation for elements by ICP-OES analysis

| Element | LOD | LOQ | Sensitivity | linearity |
| --- | --- | --- | --- | --- |
|  | (µg·L^-1^) | (µg·L^-1^) | (slope) | R² |
| Al | 12.8 | 42.7 | 31704 | 0.9999 |
| Ca | 79.2 | 264 | 6248 | 0.9999 |
| Fe | 3.91 | 13.0 | 31051 | 0.9999 |
| K | 93.4 | 311 | 1730 | 0.9941 |
| Mg | 64.3 | 214 | 1710 | 0.9995 |
| Na | 64.6 | 215 | 13980 | 0.9997 |
| P | 38.7 | 129 | 933 | 0.9999 |
| S | 129 | 431 | 173 | 0.9999 |
| Si | 66.1 | 220 | 10978 | 0.9999 |
| Pb | 27.5 | 91.7 | 1902 | 0.9998 |


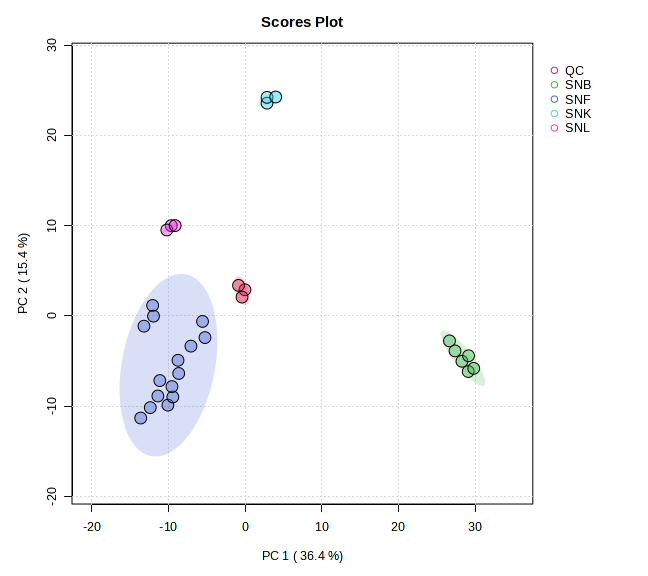

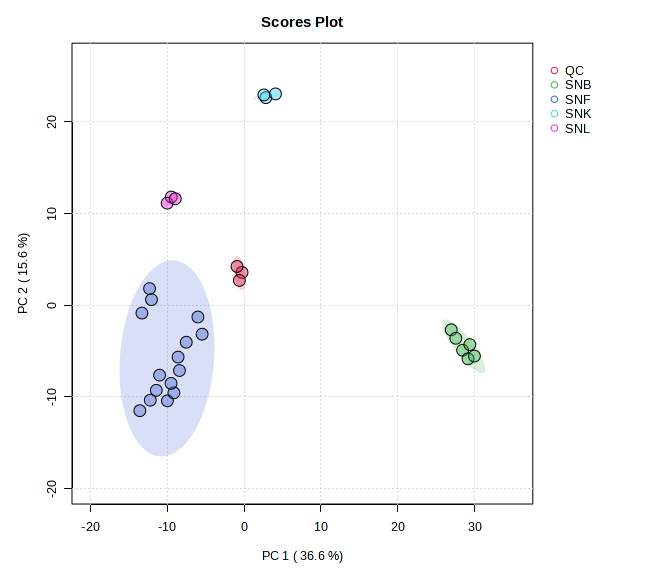

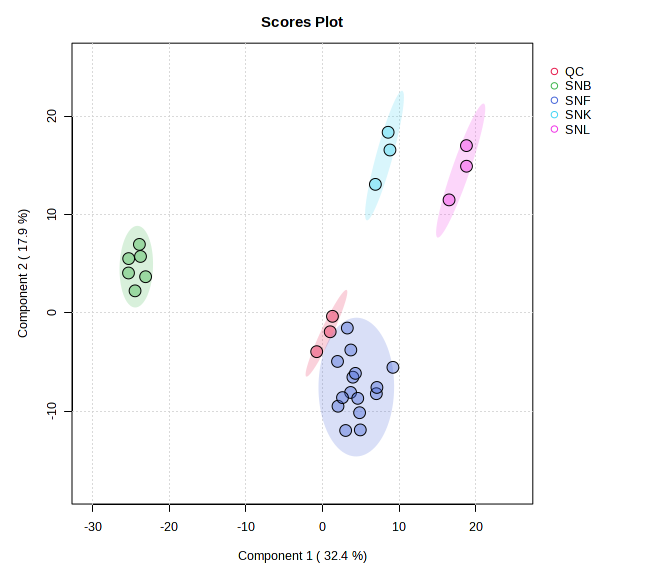

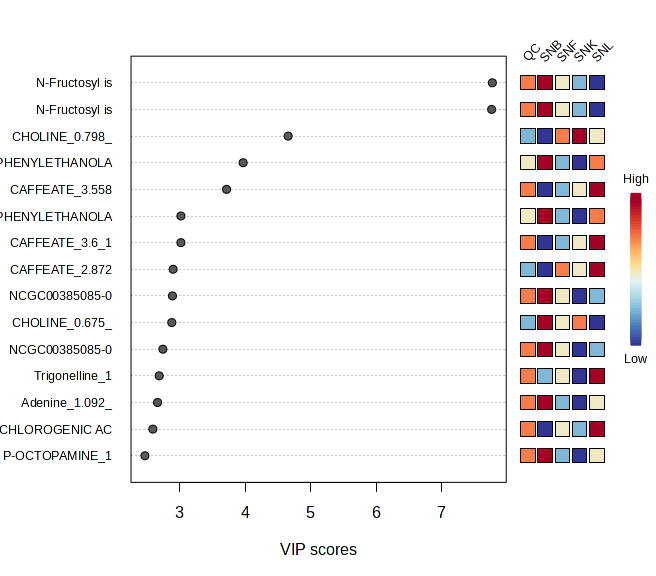

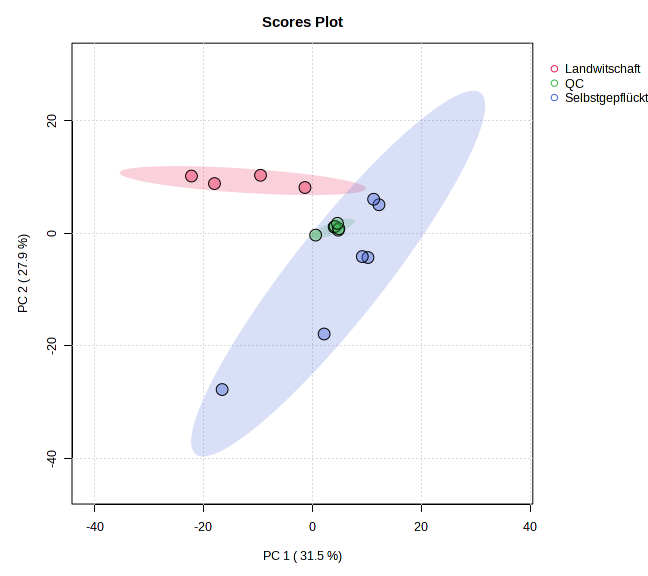

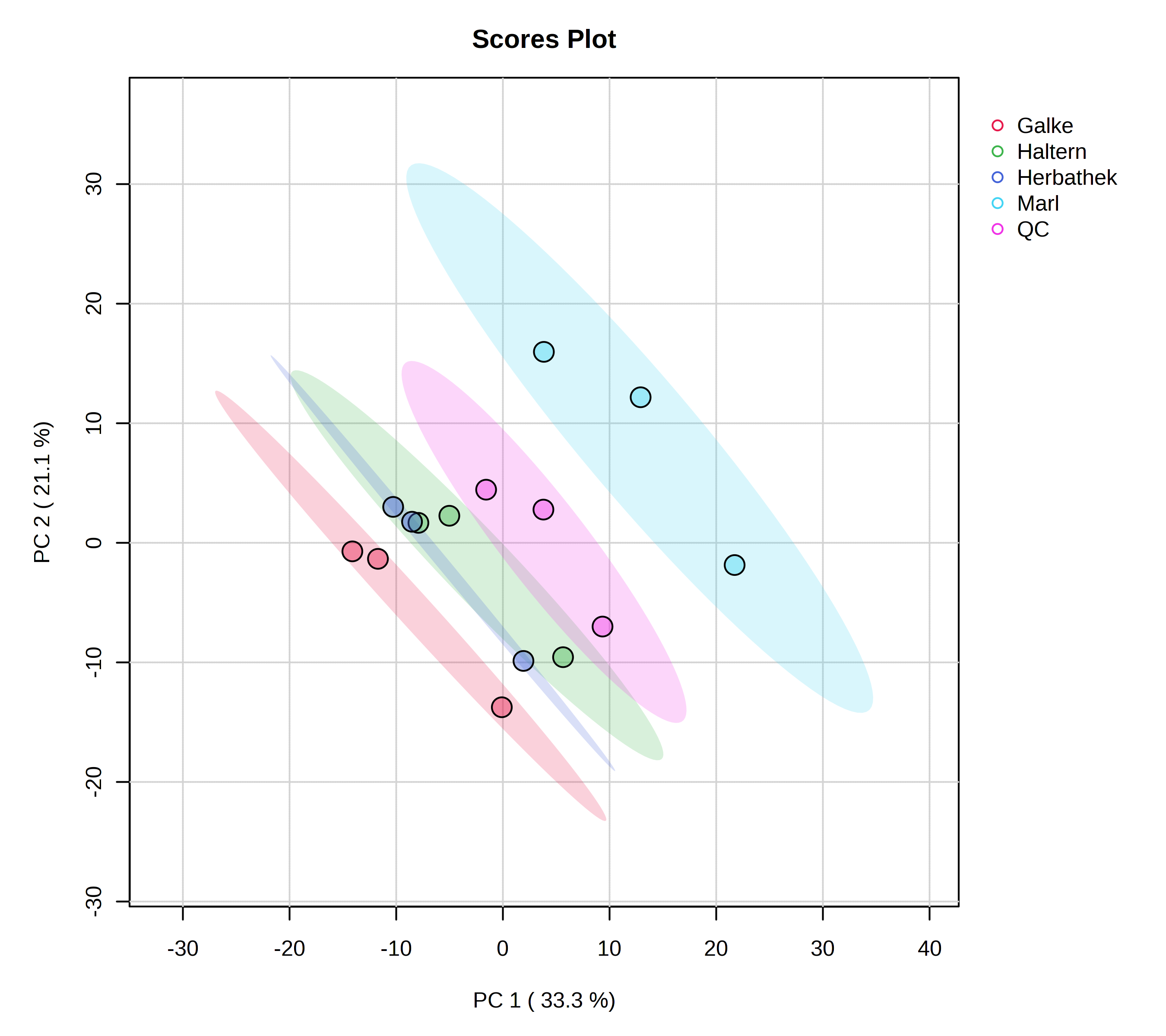

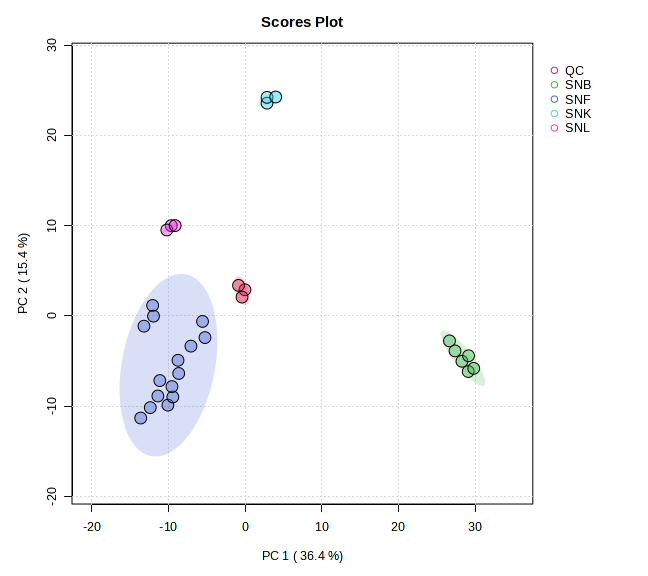

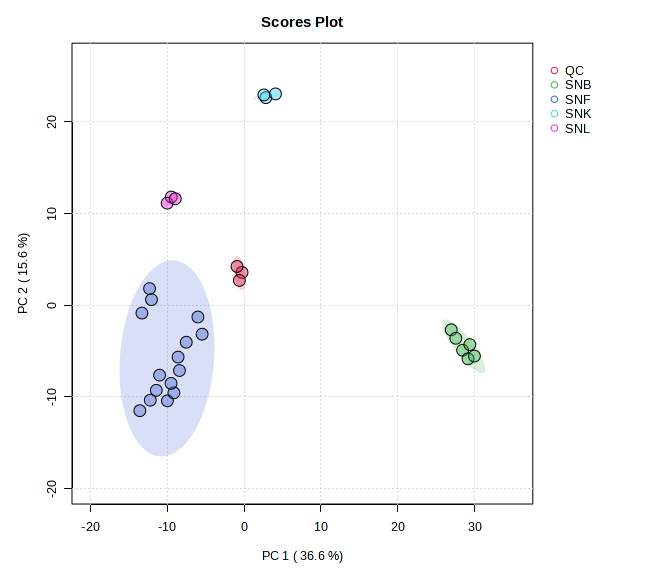

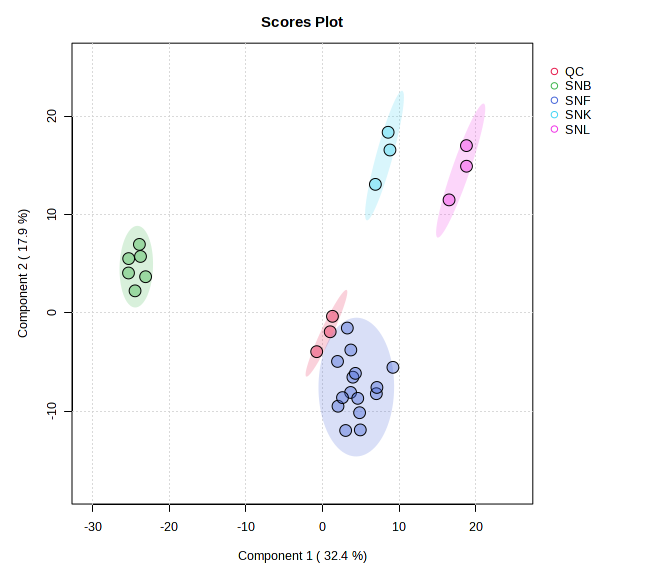

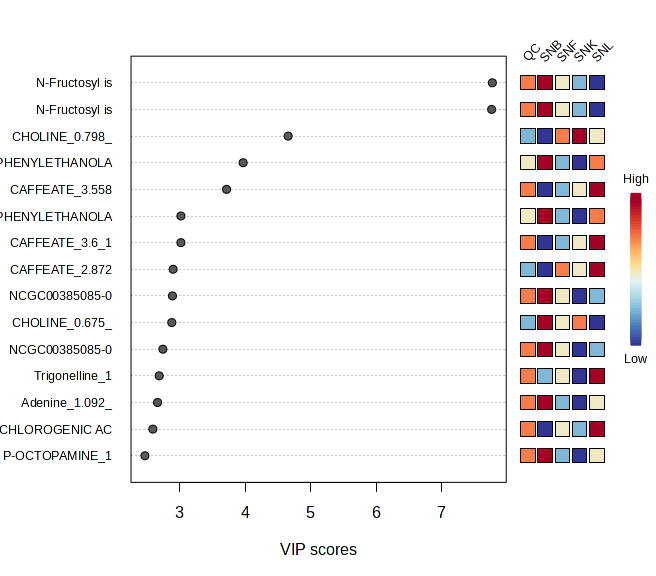

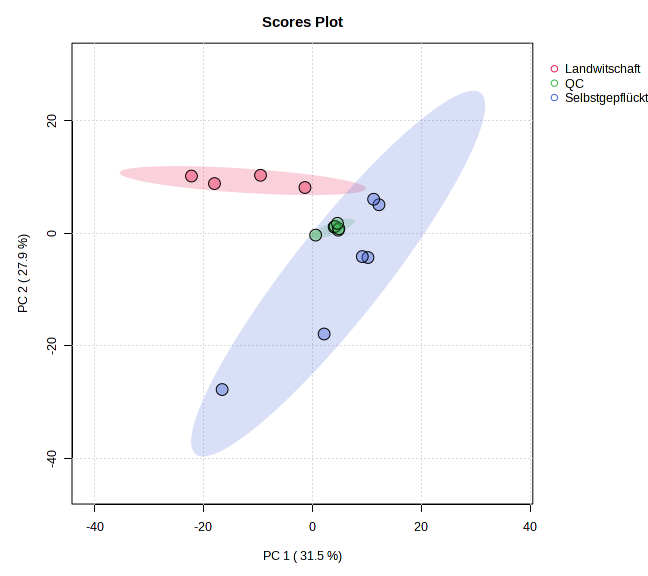


**A**

**B**

**C**

**D**

**E**

**F**

Figure S1: Comparative metabolomic profiling of S. nigra groups before (A) and after filtering (B) using PCA and PLS-DA Analyses (D) for a focus on unknown compound investigation and variable importance scores (E) for highlighting. Profiling the flowers because of the biggest sample size (C) by PCA and Nature harvested (blue) and Agricultural farmed (Red) as PCA Analysis (F) for comparison.

Table S4: Concentration of Elements within the Samples determined with Quintuplicate ICP-OES measurements

|  | Flowers | Flowers | Flowers | Flowers | Roots | Bark | Bark | Berries | Berries | Leaves |
| --- | --- | --- | --- | --- | --- | --- | --- | --- | --- | --- |
| Conc. in ppm | Germany (Haltern am See) | Bosnia | Germany (Marl) | Poland | Germany (Essen) | Germany (Essen) | Essen | Poland | Poland | Germany (Haltern am See) |
| Al | 0.17 | 0.07 | 0.13 | 0.078 | 0.10 | 0.23 | 0.075 | 0.018 | 0.039 | 0.23 |
| Ca | 6.0 | 4.5 | 7.3 | 4.6 | 8.3 | 33 | 13 | 2.8 | 2.7 | 8.9 |
| Fe | 0.14 | 0.12 | 0.15 | 0.13 | 0.13 | 0.38 | 0.074 | 0.047 | 0.083 | 0.25 |
| K | 3.6 | 3.2 | 30 | 25.8 | 5.6 | 3.2 | 8.8 | 16 | 19 | 13 |
| Mg | 4.4 | 3.5 | 3.9 | 3.0 | 4.5 | 3.8 | 3.7 | 1.8 | 1.9 | 4.2 |
| Na | 0.06 | <0.001 | <0.001 | <0.001 | 0.004 | 0.097 | <0.001 | <0.001 | <0.001 | 0.071 |
| P | 4.4 | 4.9 | 3.9 | 4.5 | 2.2 | 0.86 | 0.84 | 2.6 | 2.6 | 2.0 |
| S | 0.32 | 3.6 | 3.2 | 2.8 | 1.5 | 1.7 | 1.3 | 1.3 | 1.3 | 1.9 |
| Si | 0.29 | 0.10 | 0.24 | 0.15 | 0.16 | 0.29 | 0.13 | 0.041 | 0.076 | 0.55 |
| Mn | <0.01 | <0.01 | <0.01 | <0.01 | <0.01 | <0.01 | <0.01 | <0.01 | <0.01 | <0.01 |
| Pb | <0.01 | <0.01 | <0.01 | <0.01 | <0.01 | <0.01 | <0.01 | <0.01 | <0.01 | <0.01 |

Table S5: Detected Features listed. Sorted by level of identification.

| Compound Name | RT  (min) | Sum formula | Mass (measured) | Addukt  (Pos) | monoisotopic mass | Substanceclass | Polyphenol | Level |
| --- | --- | --- | --- | --- | --- | --- | --- | --- |
| Apigenin | 12.0 | C_15_H_10_O_5_ | 271.0607 | [M+H]^+^ | 270.0528 | Flavonoid | Yes | 1 |
| Bergapten | 11.8 | C_12_H_8_O_4_ | 217.0506 | [M+H]^+^ | 216.0423 | Coumarin | No | 1 |
| Caffeic acid | 3.6 | C_9_H_8_O_4_ | 163.0352 | [M-H_2_O+H]^+^ | 180.0423 | Cinnamic acid derivative | Yes | 1 |
| Catechin | 3.3 | C_15_H_14_O_6_ | 291.0862 | [M+H]^+^ | 290.0790 | Flavonoid | Yes | 1 |
| Chlorogenic acid | 3.6 | C_16_H_18_O_9_ | 355.1032 | [M+H]^+^ | 354.0951 | Cinnamic acid derivative | Yes | 1 |
| Coumaric acid | 3.9 | C_9_H_8_O_3_ | 147.0443 | [M-H_2_O+H]^+^ | 164.0473 | Cinnamic acid derivative | Yes | 1 |
| Coumarin | 5.9 | C_9_H_7_O_3_- | 147.0442 | [M+H]^+^ | 146.0572 | Coumarin | No | 1 |
| Curcumin | 3.4 | C_21_H_20_O_6_ | 369.1331 | [M+H]^+^ | 368.1260 | Ketone derivative | No^b^ | 1 |
| Ferulic acid | 4.7 | C_10_H_10_O_4_ | 177.0551 | [M-H_2_O+H]^+^ | 194.0579 | Cinnamic acid derivative | Yes | 1 |
| Gallic acid | 1.4 | C_7_H_6_O_5_ | 170.0284 | [M+H]^+^ | 170.0215 | Phenolic acid | Yes | 1 |
| Naringenin | 11.9 | C_15_H_12_O_5_ | 273.0771 | [M+H]^+^ | 272.0685 | Flavonoid | Yes | 1 |
| Phenylalanine | 1.8 | C_9_H_11_NO_2_ | 166.0867 | [M+H]^+^ | 165.0790 | Amine | No | 1 |
| Quercetin | 10.3 | C_15_H_10_O_7_ | 303.0509 | [M+H]^+^ | 302.0427 | Flavonoid | Yes | 1 |
| Rutin | 5.4 | C_27_H_30_O_16_ | 611.1629 | [M+H]^+^ | 610.1534 | Flavonoid | Yes | 1 |
| Sucrose | 0.7 | C_12_H_22_O_11_ | 365.1060 | [M+Na]^+^ | 342.1162 | Carbohydrate | No | 1 |
| Vanillic acid | 3.6 | C_8_H_8_O_4_ | 169.04991 | [M+H]^+^ | 168.0423 | Phenolic acid | No (Monophenol) | 1 |
| Vanillin | 4.1 | C_8_H_8_O_3_ | 153.0548 | [M+H]^+^ | 152.0473 | Phenol derivative | No (Monophenol) | 1 |
| Myricetin | 7.1 | C_15_H_10_O_8_ | 319.0437 | [M+H]^+^ | 318.0376 | Flavonoid | Yes | 1 |
|  |  |  |  |  |  |  |  |  |
| Adenine | 1.1 | C_5_H_5_N_5_ | 136.0759 | [M+H]^+^ | 135.0545 | Nucleobase | No | 2 |
| Arginine | 0.6 | C_6_H_14_N_4_O_2_ | 175.1197 | [M+H]^+^ | 174.1117 | Amino acid | No | 2 |
| CinnamicAcid | 1.8 | C_9_H_8_O_2_ | 131.0493 | [M-H_2_O+H]^+^ | 148.0524 | Cinnamic acid derivative | No | 2 |
| Dopamine | 1.1 | C_8_H_11_NO_2_ | 154.0866 | [M+H]^+^ | 153.0790 | Amine | No | 2 |
| Enoxolone | 12.6 | C_30_H_46_O_4_ | 471.3478 | [M+H]^+^ | 470.3396 | Triterpenoid | No | 2 |
| Flavovilloside | 4.3 | C_33_H_40_O_20_ | 757.2196 | [M+H]^+^ | 756.2113 | Flavonoid | Yes | 2 |
| Glutarylcarnitine | 1.2 | C_12_H_21_NO_6_ | 276.1449 | [M+H]^+^ | 275.1369 | Carnitine derivative | No | 2 |
| Guanine | 1.3 | C_5_H_5_N_5_O | 152.0569 | [M+H]^+^ | 151.0494 | Nucleobase | No | 2 |
| Isochlorogenic acid A | 5.0 | C_25_H_24_O_12_ | 499.1241 | [M-H_2_O+H]^+^ | 516.1268 | Cinnamic acid derivative | Yes | 2 |
| Kaempferol 3-O-sophoroside | 4.6 | C_27_H_30_O_16_ | 611.1614 | [M+H]^+^ | 610.1534 | Flavonoid | Yes | 2 |
| Neochlorogenic acid | 3.2 | C_16_H_18_O_9_ | 355.1027 | [M+H]^+^ | 354.0951 | Cinnamic acid derivative | Yes | 2 |
| N-Fructosyl isoleucine | 1.1 | C_12_H_23_NO_7_ | 294.1561 | [M+H]^+^ | 293.1475 | Amino acid derivative | No | 2 |
| P-Octopamine | 1.1 | C_8_H_11_NO_2_ | 136.0761 | [M-H_2_O+H]^+^ | 153.0790 | Amine | No | 2 |
| Proanthocyanidin B1 | 3.4 | C_30_H_26_O_12_ | 579.1507 | [M+H]^+^ | 578.1424 | Polyphenol | Yes | 2 |
| Quercetin 3-(6''-malonyl-glucoside) | 6.1 | C_24_H_22_O_15_ | 551.1047 | [M+H]^+^ | 550.0959 | Flavonoid | Yes | 2 |
| Quercetin-3-O-hexoside | 5.4 | C_21_H_20_O_12_ | 465.1035 | [M+H]^+^ | 464.0955 | Flavonoid | Yes | 2 |
| Tryptophan | 3.5 | C_11_H_12_N_2_O_2_ | 205.0976 | [M+H]^+^ | 204.0899 | Amino acid | No | 2 |
| Tyrosine | 1.1 | C_9_H_11_NO_3_ | 182.0814 | [M+H]^+^ | 181.0739 | Amino acid | No | 2 |
| Umbelliferone | 4.5 | C_9_H_6_O_3_ | 163.0392 | [M+H]^+^ | 162.0317 | Coumarin | No | 2 |
| Quercetin diglucoside | 4.2 | C_27_H_30_O_17_ | 627.1565 | [M+H]^+^ | 0.0000 | Flavonoid | Yes | 2 |
|  |  |  |  |  |  |  |  |  |
| (-)-epicatechin | 3.4 | C_15_H_14_O_6_ | 291.0868 | [M+H]^+^ | 290.0790 | Flavonoid | Yes | 3 |
| (22E)-3alpha,12alpha-Dihydroxy-5beta-chol-22-en-24-oic Acid | 14.5 | C_24_H_38_O_4_ | 803.5467 | [2M+Na] | 390.2770 | Steroid acid | No | 3 |
| (2S)-4-[(1R)-2,6,6-Trimethyl-4-oxo-2-cyclohexen-1-yl]-2-butanyl β-D-glucopyranoside | 5.6 | C_19_H_32_O_7_ | 373.2227 | [M+H]^+^ | 372.2148 | Glycoside | No | 3 |
| 1,4-Dihydroxy-16-heptadecen-2-yl acetate | 12.7 | C_19_H_36_O_4_ | 351.2512 | [M+Na]^+^ | 328.2614 | Polyhydroxy fatty acid ester | No | 3 |
| 10-Hydroxydecanoate | 4.4 | C_10_H_19_O_3_ | 153.1275 | [M-2H_2_O+H]^+^ | 187.1340 | Fatty acid derivative | No | 3 |
| 1-palmitoyl-2-hydroxy-sn-glycero-3-phosphoethanolamin | 13.0 | C_21_H_44_NO_7_P | 454.2943 | [M+H]^+^ | 453.2856 | Phospholipid | No | 3 |
| 1-Stearoyl-sn-glycero-3-phosphocholine | 14.3 | C_26_H_54_NO_7_P | 524.3724 | [M+H]^+^ | 523.3638 | Phospholipid | No | 3 |
| 2-(hydroxymethyl)-6-[4-[(2S,3S)-3-(hydroxymethyl)-5-[(E)-3-hydroxyprop-1-enyl]-7-methoxy-2,3-dihydro-1-benzofuran-2-yl]-2-methoxyphenoxy]oxane-3,4,5-triol | 4.4 | C_26_H_32_O_11_ | 503.1920 | [M-H_2_O+H]^+^ | 520.1945 | Glycoside | No | 3 |
| 2-Oxindole | 2.0 | C_8_H_7_NO | 134.0601 | [M+H]^+^ | 133.0528 | Indole derivative | No | 3 |
| 2-Phenylglycine | 3.6 | C_8_H_9_NO_2_ | 135.0442 | [M-NH_4_+2H]^+^ | 151.0633 | Amino acid derivative | No | 3 |
| 3-Hydroxy-11-ursen-28,13-olid | 12.5 | C_30_H_46_O_3_ | 437.3428 | [M-H_2_O+H]^+^ | 454.3447 | Triterpenoid | No | 3 |
| 3-Hydroxy-13,28-epoxyurs-11-en-28-one | 12.5 | C_30_H_46_O_3_ | 437.3428 | [M-H_2_O+H]^+^ | 454.3447 | Triterpenoid | No | 3 |
| 3-p-Coumaroylquinic acid | 3.9 | C_16_H_18_O_8_ | 339.1082 | [M+H]^+^ | 338.1002 | Cinnamic acid derivative | Yes | 3 |
| 4,9-dimethoxyfuro[3,2-g]chromen-7-one | 11.7 | C_13_H_10_O_5_ | 247.0615 | [M+H]^+^ | 246.0528 | Coumarin derivative | No | 3 |
| 4-Guanidino-butanoate | 0.8 | C_5_H_11_N_3_O_2_ | 146.0925 | [M+H]^+^ | 145.0851 | Amino acid derivative | No | 3 |
| 4-O-feruloyl-D-quinic acid | 4.2 | C_17_H_20_O_9_ | 369.1184 | [M+H]^+^ | 368.1107 | Cinnamic acid derivative | Yes | 3 |
| 5-Aminopentanoate | 13.4 | C_5_H_11_NO_2_ | 100.0758 | [M-H_2_O+H]^+^ | 117.0790 | Amino acid derivative | No | 3 |
| Adenosine | 1.1 | C_10_H_13_N_5_O_4_ | 268.1044 | [M+H]^+^ | 267.0968 | Nucleoside | No | 3 |
| Alpha-Tocopherol (Vitamin E) | 15.7 | C_29_H_50_O_2_ | 429.3744 | [M-H_2_O+H]^+^ | 430.3811 | Vitamin | No | 3 |
| Antimycin A2 | 16.3 | C_27_H_38_N_2_O_9_ | 535.2728 | [M+H]^+^ | 534.2577 | Antibiotic | No | 3 |
| Archangelicine | 12.7 | C_24_H_26_O_7_ | 444.2026 | [M+NH_4_]^+^ | 426.1679 | Coumarin derivative | No | 3 |
| Benzoic acid | 1.1 | C_7_H_6_O_2_ | 123.0443 | [M+H]^+^ | 122.0368 | Phenolic acid | No | 3 |
| Choline | 0.8 | C_5_H_14_CINO | 104.1075 | [M+H]^+^ | 104.1075 | Quaternary ammonium compound | No | 3 |
| Citrate | 0.9 | C_6_H_5_O_7_ | 190.0042 | [M+H]^+^ | 189.0035 | Carboxylic acid derivative | No | 3 |
| Cynarine | 7.1 | C_25_H_24_O_12_ | 517.1345 | [M+H]^+^ | 516.1268 | Cinnamic acid derivative | Yes | 3 |
| Geranic acid | 5.1 | C_10_H_16_O_2_ | 169.1227 | [M+H]^+^ | 168.1150 | Terpenoid | No | 3 |
| Guanosine | 1.3 | C_10_H_13_N_5_O_5_ | 284.0994 | [M+H]^+^ | 283.0917 | Nucleoside | No | 3 |
| imperatorin | 12.1 | C_16_H_14_O_4_ | 271.0974 | [M+H]^+^ | 270.0892 | Coumarin derivative | No | 3 |
| Isorhamnetin | 4.8 | C_16_H_12_O_7_ | 317.0661 | [M+H]^+^ | 316.0583 | Flavonoid | Yes | 3 |
| isorhamnetin-3-O-glucoside | 4.8 | C_22_H_22_O_12_ | 479.1188 | [M+H]^+^ | 478.1111 | Flavonoid | Yes | 3 |
| Kaempferol | 4.6 | C_15_H_10_O_6_ | 287.0555 | [M+H]^+^ | 286.0477 | Flavonoid | Yes | 3 |
| Kaempferol-3-O-rutinoside | 6.5 | C_27_H_30_O_15_ | 595.1677 | [M+H]^+^ | 594.1585 | Flavonoid | Yes | 3 |
| kaempferol-7-O-hexoside | 3.9 | C_21_H_20_O_11_ | 449.1089 | [M+H]^+^ | 448.1006 | Flavonoid | Yes | 3 |
| Loliolide | 4.7 | C_11_H_16_O_3_ | 197.1176 | [M+H]^+^ | 196.1099 | Monoterpene lactone | No | 3 |
| L-Valine | 0.9 | C_5_H_11_NO_2_ | 118.0862 | [M+H]^+^ | 117.0790 | Amino acid | No | 3 |
| Morin | 6.1 | C_15_H_10_O_7_ | 303.0503 | [M+H]^+^ | 302.0427 | Flavonoid | Yes | 3 |
| Narcissin | 6.9 | C_28_H_32_O_16_ | 625.1782 | [M+H]^+^ | 624.1690 | Flavonoid | Yes | 3 |
| Oroselol | 12.8 | C_14_H_12_O_4_ | 227.0714 | [M-H_2_O+H]^+^ | 244.0736 | Sesquiterpene lactone | No | 3 |
| Osthol | 12.2 | C_15_H_16_O_3_ | 245.1184 | [M+H]^+^ | 244.1099 | Coumarin derivative | No | 3 |
| palmitic Acid | 12.9 | C_16_H_32_O_2_ | 255.2520 | [M+H]^+^ | 256.2402 | Fatty acid | No | 3 |
| P-Anisaldehyde | 6.0 | C_8_H_8_O_2_ | 137.0601 | [M+H]^+^ | 136.0524 | Aromatic aldehyde | No | 3 |
| p-Coumaraldehyde | 1.8 | C_9_H_8_O_2_ | 149.0598 | [M+H]^+^ | 148.0524 | Phenolic aldehyde | No | 3 |
| Pheophytin A | 18.3 | C_55_H_74_N_4_O_5_ | 871.5771 | [2M+H]^+^ | 870.5659 | Chlorophyll derivative | No | 3 |
| Proline | 0.8 | C_5_H_9_NO_2_ | 116.0707 | [M+H]^+^ | 115.0633 | Amino acid | No | 3 |
| Pyridoxal | 12.4 | C_8_H_9_NO_3_ | 150.0555 | [M-H_2_O+H]^+^ | 167.0582 | Vitamin | No | 3 |
| Pyrogallol | 1.9 | C_6_H_6_O_3_ | 127.0390 | [M+H]^+^ | 126.0317 | Phenol | Yes | 3 |
| Rhamnetin 3-sophoroside | 4.8 | C_28_H_32_O_17_ | 641.1721 | [M+H]^+^ | 640.1639 | Flavonoid | Yes | 3 |
| Rosmarinic acid | 7.2 | C_18_H_16_O_8_ | 163.0393 | [M+H-C_9_H_10_O_5_]^+^ | 360.0845 | Phenolic acid | Yes | 3 |
| Thymine | 2.0 | C_5_H_6_N_2_O_2_ | 127.0502 | [M+H]^+^ | 126.0429 | Nucleobase | No | 3 |
| Trigonelline | 1.0 | C_7_H_7_NO_2_ | 138.0631 | [M+H]^+^ | 137.0477 | Alkaloid | No | 3 |

Table S6: Concentration of level 1 identified compounds within the samples calculated by triplicate external calibration.

| Name | LOD | LOQ | Flower Marl | Flower Haltern | Flower Herbathek | Flower Galke | Leaves Haltern | Bark Essen | Berries Galke | Berries Herbathek | Roots Essen |
| --- | --- | --- | --- | --- | --- | --- | --- | --- | --- | --- | --- |
|  | (µM] | (µM) | (µM) | (µM) | (µM) | (µM) | (µM) | (µM) | (µM) | (µM) | (µM) |
| Apigenin | 0.065 | 0.218 | 0.12 | 0.32 | 0.3 | 0.27 | <LOD | <LOD | <LOD | <LOD | <LOD |
|  |  |  | ±0.017 | ±0.054 | ±0.071 | ±0.059 |  |  |  |  |  |
| Bergapten | 0.002 | 0.007 | 0.01 | 0.04 | <LOD | <LOD | 0.02 | 0.02 | <LOD | <LOD | <LOD |
|  |  |  | ±0.002 | ±0.003 |  |  | ±0.002 | ±0.003 |  |  |  |
| Caffeic acid | 0.095 | 0.315 | 34.03 | 20.8 | 28.05 | 31 | 31.8 | 8.56 | 2.68 | 1.24 | <LOD |
|  |  |  | ±2.64 | ±3.59 | ±1.29 | ±1.02 | ±1.15 | ±0.182 | ±0.091 | ±0.09 |  |
| Catechin hydrate | 0.04 | 0.134 | 0.83 | 0.55 | 0.36 | 0.24 | <LOD | 0.41 | <LOD | <LOD | <LOD |
|  |  |  | ±0.134 | ±0.141 | ±0.089 | ±0.085 |  | ±0.011 |  |  |  |
| Chlorogenic acid | 1.178 | 3.927 | 231 | 136 | 193 | 200 | 207 | 56.4 | 19.6 | 9.65 | <LOD |
|  |  |  | ±25.2 | ±11.9 | ±9.74 | ±6.95 | ±19.7 | ±5.88 | ±1.12 | ±0.398 |  |
| Coumaric acid | 0.261 | 0.869 | 116 | 70.1 | 71.43 | 57.8 | 27.8 | 1.08 | 2.04 | <LOD | <LOD |
|  |  |  | ±4.03 | ±6.16 | ±6.81 | ±6.82 | ±2.43 | ±1.04 | ±0.267 |  |  |
| Coumarin | 0.036 | 0.121 | 0.08 | 0.09 | 0.27 | 0.14 | 0.06 | 0.14 | <LOD | <LOD | <LOD |
|  |  |  | ±0.022 | ±0.046 | ±0.06 | ±0.053 | ±0.005 | ±0.021 |  |  |  |
| Curcumin | 0.01 | 0.035 | <LOD | <LOD | <LOD | <LOD | 0.09 | 0.03 | <LOD | <LOD | <LOD |
|  |  |  |  |  |  |  | ±0.02 | ±0.012 |  |  |  |
| Ferulic acid | 0.137 | 0.457 | 0.51 | <LOD | 0.69 | 0.98 | 1.56 | 2.52 | <LOD | <LOD | <LOD |
|  |  |  | ±0.253 |  | ±0.195 | ±0.423 | ±2.22 | ±0.404 |  |  |  |
| Gallic acid | 0.557 | 1.855 | <LOD | <LOD | <LOD | <LOD | <LOD | <LOD | 0.66 | 0.87 | <LOD |
|  |  |  |  |  |  |  |  |  | ±0.08 | ±0.047 |  |
| Naringenin | 0.004 | 0.014 | 3.52 | 3.36 | 9.45 | 7.43 | 0.35 | <LOD | <LOD | <LOD | <LOD |
|  |  |  | ±0.599 | ±0.38 | ±0.208 | ±0.449 | ±0.038 |  |  |  |  |
| Phenylalanine | 0.084 | 0.279 | 27.1 | 51.2 | 43.36 | 38.6 | 37.8 | 17.93 | 31.8 | 44.9 | <LOD |
|  |  |  | ±11.9 | ±21.1 | ±16.0 | ±15.8 | ±3.58 | ±4.55 | ±1.60 | ±3.21 |  |
| Rutin | 0.038 | 0.125 | 173 | 180 | 191 | 186 | 189 | 0.97 | 64.1 | 53.6 | <LOD |
|  |  |  | ±16.3 | ±17.6 | ±19.6 | ±21.7 | ±17.1 | ±0.899 | ±6.08 | ±3.75 |  |
| Tryptophan | 0.034 | 0.112 | 16.31 | 31.24 | 24.18 | 26.92 | 31.4 | 26.86 | 5.46 | 6.02 | 0.06 |
|  |  |  | ±3.099 | ±3.018 | ±1.575 | ±1.443 | ±1.72 | ±2.96 | ±0.362 | ±0.307 | ±0.114 |
| Vanillic acid | 0.002 | 0.006 | 0.003 | 0.066 | 0.005 | 0.065 | 0.063 | <LOD | <LOD | <LOD | <LOD |
|  |  |  | ±0.001 | ±0.001 | ±0.001 | ±0.002 | ±0.001 |  |  |  |  |
| Vanillin | 0.019 | 0.063 | 0.03 | 0.05 | 0.03 | 0.07 | 0.05 | 0.37 | 0.08 | 0.07 | 0.04 |
|  |  |  | ±0.004 | ±0.003 | ±0.003 | ±0.005 | ±0.007 | ±0.054 | ±0.008 | ±0.002 | ±0.039 |
